# Supplementary material for: Advanced Clinical Practitioners in Primary Care in the UK: A Qualitative Study of Workforce Transformation
Source: Int J Environ Res Public Health. 2020 Jun 23;17(12):4500. doi: 10.3390/ijerph17124500 (PMC7344450; doi:10.3390/ijerph17124500)
Supplement: Supplementary file 1 [file ijerph-17-04500-s001.zip › SUPPLEMENTARY FILE 1 (Information Sheet).docx]

# SUPPLEMENTARY FILE 1: PARTICIPANT INFORMATION SHEET

**
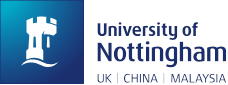
**

**Participant Information Sheet**

**(Final version 1.0: 04.02.19)**

**Title of Study: evaluation of the role of the advanced clinical practitioner in primary care across the East Midlands**

**Name of Chief Investigator**: Dr Holly Blake

**Local Researcher(s):** Sarah Greaves, Ruth Pearce (Co-I), Catrin Evans (Co-I)

We would like to invite you to take part in our evaluation study. Before you decide, we would like you to understand why the evaluation is being done and what it would involve for you. One of our team will go through the information sheet with you and answer any questions you have. Talk to others about the study if you wish. Ask us if there is anything that is not clear.

**What is the purpose of the study?**

The purpose of the study is to provide an in-depth understanding of the contribution the advanced clinical practitioner (ACP) in primary care practice, including impacts on clinical care, the workforce and the wider organisation.

**Why have I been invited?**

You are being invited to take part because you work in a practice that has an ACP appointed. We are inviting healthcare professionals (GPs, practice managers and ACPs) to take part from a range of practices in Derbyshire and Nottinghamshire.

**Do I have to take part?**

It is up to you to decide whether or not to take part. If you do decide to take part, you will be given this information sheet to keep and be asked to sign a consent form. If you decide to take part you are still free to withdraw at any time and without giving a reason.

**What will happen to me if I take part?**

If you take part in this study, you will be invited to take part in a telephone interview with one of our local researchers, to discuss the ACP role. We will record this via a dictaphone with your permission. The interview will be informal in nature, lasting approximately 20-30 minutes depending on the answers you provide.

**Expenses and payments**

Participants will not be paid to participate in the study.

**What are the possible disadvantages and risks of taking part?**

We do not foresee there to be any risks from taking part in this study. There may be some inconvenience associated with being interviewed but this is for the benefit of future service provision within primary care practice.

**What are the possible benefits of taking part?**

The information we get from this study may help to improve our understanding of the ACP role, alongside any barriers and enablers to implementing the role in practice, which can ultimately lead to future recommendations for clinical care.

**What happens when the research study stops?**

When the study stops, the data gathered will be analysed so that we can draw conclusions and make service recommendations. There will be an option to be informed about the outcome of the study, and you will be able to indicate on your consent form if you would like to receive this.

**What if there is a problem?**

If you have a concern about any aspect of this study, you should ask to speak to the researchers who will do their best to answer your questions. The researchers’ contact details are given at the end of this information sheet. If you do not receive a satisfactory answer then you can contact the Chief Investigator of the project.

**Will my taking part in the study be kept confidential?**

We will follow ethical and legal practice and all information about you will be handled in confidence.

If you join the study, we will use information collected from you during the course of the evaluation. This information will be kept strictly confidential, stored in a secure and locked office, and on a password-protected database at the University of Nottingham. The data collected for the study will be looked at and stored by authorised persons from the University of Nottingham who are organising the project. They may also be looked at by authorised people from regulatory organisations to check that the study is being carried out correctly. We will keep anonymised data securely for 7 years. After this time your data will be disposed of securely. During this time all precautions will be taken by all those involved to maintain your confidentiality, only members of the research team given permission by the data custodian (Chief Investigator) will have access to your personal data. We will aim to write up the anonymised study findings into a report. Although what you say to us is confidential, should you disclose anything to us which we feel puts you or anyone else at any risk, we may feel it necessary to report this to the appropriate persons.

**What will happen if I don’t want to carry on with the study?**

Your participation is voluntary and you are free to withdraw at any time, without giving any reason, and without your legal rights being affected. If you withdraw we will no longer collect any information from you but we will retain the information about you that we have already obtained and this will be used for our evaluation analysis. We will use the minimum personally-identifiable information possible.

**What will happen to the results of the research study?**

The results of the evaluation will be written up as a report and will be published in academic journals. It may be presented locally to other clinical teams and at conferences so that others can learn from the experiences and conclusions drawn from the study.

**Who is organising and funding the research?**

This evaluation is being organised by the University of Nottingham and the project has received funding from the HEE.

**Who has reviewed the study?**

This study has been discussed with the University of Nottingham RGS Team and has been classed as service evaluation.

Further information and contact details

**Researcher:**  Sarah Greaves

School of Health Sciences,
Queen’s Medical Centre
University of Nottingham, UK
Derby Road, NG7 2HA.
Email: [mszsjg@exmail.nottingham.ac.uk](mailto:mszsjg@exmail.nottingham.ac.uk)

**Chief Investigator:** Dr Holly Blake (Associate Professor of Behavioural Science)
School of Health Sciences
University of Nottingham
B Floor, Queen's Medical Centre
Nottingham, NG7 2HA
Phone: +44 (0) 115 8231049
Email: [Holly.Blake@nottingham.ac.uk](mailto:Holly.Blake@nottingham.ac.uk)
