# Supplementary material for: Advanced Clinical Practitioners in Primary Care in the UK: A Qualitative Study of Workforce Transformation
Source: Int J Environ Res Public Health. 2020 Jun 23;17(12):4500. doi: 10.3390/ijerph17124500 (PMC7344450; doi:10.3390/ijerph17124500)
Supplement: Supplementary file 1 [file ijerph-17-04500-s001.zip › SUPPLEMENTARY FILE 4 (PM & GP Interview Guide).docx]

# SUPPLEMENTARY FILE 4: INTERVIEW GUIDE FOR GPS AND PRACTICE MANAGERS

**Evaluation of the role of the advanced clinical practitioner in primary care across**

**the East Midlands**

Project Team: Holly Blake, Ruth Pearce, Catrin Evans

**Interview guide: GPs/Practice Managers**

General introduction / ice breaker

Recap purpose of the interview

- I’d like to ask you about the ACP role. The interview is confidential and will inform future practice. Please be honest in your responses.
- The interview will be recorded, with permission, and then will be typed up to use as anonymised data for evaluation purposes.
- If there’s any questions you do not want to answer then that’s ok and if at any time you want the interview to stop that’s fine too.
- If it’s ok with you I will turn the recorder on now and everything we talk about from now on will be recorded.
- *Are you happy for me to turn the tape on?*

**Start recording: *** Obtain verbal consent to participate on tape *****

**Background to the practice**

**Can you tell me about the practice that you work in?**

*Prompts:*

- What is the size of the practice?
- What is the setting (e.g. rural, urban, area – affluent or deprivation)?
- How many GPs at the practice?

**What was the reason for appointing an ACP at your practice?**

**Understanding of ACP role**

**Can you tell me about the ACP role in your practice, with regards their appointment, training, and contribution to the practice?**

*Prompts:*

- What is the professional background of the ACP?
- What training do they have for the ACP role?
- How long have they been in post?
- What is the nature and type of activities the ACP undertakes?
- How many patients do they see?
- Number of practice staff supported by ACP?

**Evaluation of ACP role**

**What are your general experiences of having an ACP within your practice?**

*Prompts:* level of satisfaction, experiences, patient and team engagement with ACP, acceptability, perceived usefulness, facilitators of the role, contributions of ACP

**What are the overall impacts on the practice of having an ACP in post?**

*Prompts:* clinical outcomes, service accessibility, impacts on team workload and workflow, patient waiting times, enhanced services, choice of clinician, team support for ACP & training

**What are the impacts on you as an individual of having an ACP in post?**

*Prompts:* individual support for ACP & training of ACP, impacts on individual workload, employee wellbeing, stress levels, work engagement, job satisfaction

**What do you see to be the main challenges of having an ACP within your practice?**

*Prompts:* resources*,* costs, training, workload, barriers to access, acceptability, team or patient understanding of role

**Would you recommend the ACP role to other practices?**

*Prompts: Yes/No, why?*

**Is there anything about the ACP role that you would change?**

*Prompts:* suggestions for the future, training and costs of implementation, professional background of ACP, contributions to practice and workflow, information needs

- Is there anything else you would like to say about the ACP role?
- Do you have any questions for me?
- Do you have any concerns or queries before I turn off the tape?

Thank you for answering these questions.

** TURN OFF THE DICTAPHONE **

**END OF INTERVIEW.**
