# Supplementary figures and images for: Advanced Clinical Practitioners in Primary Care in the UK: A Qualitative Study of Workforce Transformation
Source: Int J Environ Res Public Health. 2020 Jun 23;17(12):4500. doi: 10.3390/ijerph17124500 (PMC7344450; doi:10.3390/ijerph17124500)

**SUPPLEMENTARY FILE 2: CONSENT FORM**

**
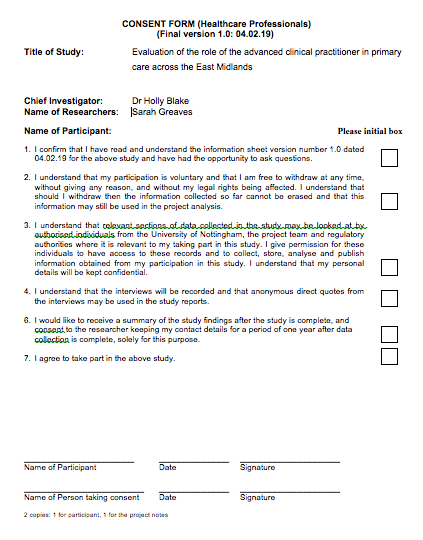
**

Supplement: Supplementary file 1 [file ijerph-17-04500-s001.zip › SUPPLEMENTARY FILE 2 (Consent Form).docx]
